# Adherence to antihypertensive treatment and associated factors among patients on follow up at University of Gondar Hospital, Northwest Ethiopia

**DOI:** 10.1186/1471-2458-12-282

**Published:** 2012-07-12

**Authors:** Abere Dessie Ambaw, Getahun Asres Alemie, Solomon Meseret W/Yohannes, Zelalem Birhanu Mengesha

**Affiliations:** 1 College of Medicine and Health Sciences, University of Gondar, Gondar, P.O.Box 196, Ethiopia; 2 Department of Epidemiology and Biostatistics, Institute of Public Health, University of Gondar, Gondar, P.O.Box 196, Ethiopia; 3 Department of Reproductive Health, Institute of Public Health, University of Gondar, Gondar, P.O.Box 196, Ethiopia

## Abstract

****Background**:**

Hypertension is an overwhelming global challenge with high morbidity and mortality rates. The prevalence of HTN is estimated to be 6% in Ethiopia and 30% in Addis Ababa. Poor adherence is associated with bad outcome of the disease and wastage of healthcare resources. In Ethiopia, particularly in the study area little is known about treatment adherence and associated factors. Therefore this study aimed to assess adherence to antihypertensive therapy and associated factors among HTN patients on follow up at University of Gondar Referral Hospital.

****Method**:**

Institution based cross sectional study was conducted. Systematic sampling technique was used to select 384 participants. A structured standard questionnaire was used after some modifications. Morisky Medication Adherence Scale was used for labeling patients as adherent or non-adherent. Data were entered and analyzed using SPSS version 16.

****Results**:**

More than half (64.6 %) of the study participants were found to be adherent to their treatment. Sex (AOR = 0.48, 95%CI = 0.28, 0.82), knowledge about HTN and its treatment (AOR = 6.21, 95%CI = 3.22, 11.97), distance from the hospital (AOR = 2.02, 95% CI =1.19-3.43) and co morbidity (AOR = 2.5, 95%CI = 1.01, 6.21) variables were found significantly associated with treatment adherence.

****Conclusion**:**

Only 64.6% of the study subjects were found to be adherent to their treatment. Factors such as sex, distance from the hospital, number of co morbidities, Knowledge about HTN and its treatment were associated with adherence behavior of patients. Early diagnosis and management of co morbidities, adherence counseling and patient education about the disease and its treatment are important to improve adherence status of patients.

## **Background**

Hypertension (HTN), or high blood pressure (systolic blood pressure ≥140 mmHg and diastolic blood pressure ≥90 mmHg) is an overwhelming global challenge which ranks third as a cause of disability-adjusted life-year [1]. Hypertension causes 7.1 million premature deaths each year worldwide and accounts for 13% of all deaths globally [2]. Analysis of the global burden of hypertension revealed that over 26% of the world's adult population had hypertension in 2000 [3]. In developing countries, its morbidity and mortality are increasing from time to time due to a change in life style and sedentary life. In Africa, 15% of the population has hypertension [4]. Although there is shortage of extensive data, 6% of the Ethiopian population has been estimated to have HTN. Approximately 30% of adults in Addis Ababa have hypertension above 140/90 mmHg or reported use of anti-hypertensive medication [5].

There are effective medical therapies for hypertension management. However, only 37% of hypertensive patients in a 2003-2004 survey were reported to have their blood pressure controlled [6]. The problem of non-adherence to medical treatment remains a challenge for the medical professions and social scientists. As a result, substantial numbers of patients do not get the maximum benefit of medical treatment, resulting in poor health outcomes, lower quality of life and increased health care costs. In spite of many advances made in adherence research, non-adherence rates have remained nearly unchanged in the last decades [1,7,8].

Poor adherence to anti-hypertensive therapy is one of the biggest obstacles in therapeutic control of high blood pressure [6]. It also compromises the efforts of the health care system, policy makers and health care professionals in improving the health of populations. Failure to adhere causes medical and psychological complications of the disease, reduces patients’ quality of life, wastes health care resources and erodes public confidence in health systems [9].

Poor adherence to anti-hypertensive therapy is usually associated with bad outcome of the disease and wastage of limited health care resources. In Ethiopia, particularly in the study area, little is known about the adherence status and associated factors. Therefore, the aim of this study was to assess the adherence status and associated factors to antihypertensive therapy among patients on follow up at University of Gondar Hospital.

## **Methods**

Institution based cross-sectional survey was done at University of Gondar teaching Hospital from April to August 2011. The University of Gondar Teaching Hospital is located in Gondar Town, Amhara National Regional State, Ethiopia. The Town consisted of one central referral Hospital and three health centers. Gondar University Hospital is a teaching as well as regional referral Hospital under ministry of education. The situation in Ethiopia is that most of the Hospitals where Universities are located are teaching referral Hospitals. The referral Hospital is meant to serve 5 million people as per the four tier system of the National Ministry of Health. Chronic illness care is one of the services the Hospital provides to the population both within and outside of Gondar Town. Provision of treatments for hypertensive patients is one of the chronic illnesses included in the chronic illness care. There were 968 patients on antihypertensive treatment at the chronic illness follow-up care. The study was conducted among hypertensive patients of age 18 years and above receiving antihypertensive treatment as outpatients at the University of Gondar Referral Hospital. A single population proportion formula, [n = (Z α/2)^2^ p (1-p)], was used to estimate the sample size. The following assumptions were made: since no previous data, proportion was taken as 50% (p = 0.5), 95% confidence interval, margin of error 5% (d = 0.05). Computing with the above formula gives a total sample size of 384. Every other 2 patients were interviewed when they come for follow up at the chronic care clinic using systematic random sampling technique from the registration list of patients.

### **Controlled hypertension**

Average BP reading less than 140/90 mmHg at the time of data collection irrespective of measurements at other times.

### **Knowledgeable**

Respondents who scored above mean for the knowledge questions.

### **Good patient-provider relationship**

Participants who scored mean and above for questions prepared on patient provider relationship in the treatment and care of HTN patients otherwise not. A standard pretested questionnaire supplemented with patient medical record review to counter check the clinical data reports was used. Data were collected by trained nurses working in the other wards of the hospital. Additional two nurses were assigned to supervise the data collection process. Morisky Medication Adherence Scale (**MMAS)**, a 4-item questionnaire with a high reliability and validity, which has been particularly useful in chronic conditions such as hypertension was used [10]. The scale is based on patient`s self response [11]. The total score ranges from 1 to 5. During analysis, a cut-off value of MMAS mean score > =3 and <3 were used for labeling patients as adherent or non-adherent respectively [12].

After participants are interviewed, two sitting BP measurements were taken in the right arm with pre-tested mercury/aneroid sphygmomanometers and stethoscopes approximately 2 min apart and the average of the two readings were used to determine the BP level. The cuff size was 23 × 12.5 cm. For all readings Korotkoff phases I and V were used to establish the levels of systolic and diastolic blood pressure, respectively.

The data were entered and analyzed using SPSS version 16 statistical package. Data cleaning was performed to check for accuracy, consistency and there were no missed values during entry. Frequencies, proportions and summary statistics were used to describe the study population in relation to relevant variables. Bivariate and multivariate analyses were carried out to see the putative associations of each independent variable with the dependent variable. Odds ratio and 95% confidence interval were also used to identify the presence and strength of association.

Ethical approval from the institutional review board of the University of Gondar was obtained. Permission to conduct the study in follow-up unit was obtained from the medical director’s office of the hospital. Each study participant was adequately informed about the purpose, method, anticipated benefit and risk of the study by the data collectors. Verbal consent was obtained from study participants and anonymity was maintained to ensure confidentiality.

## **Results**

### **Socio-demographic characteristics**

A total of 384 study participants were interviewed making the response rate 100%. The mean age of respondents was 56.9 years with a standard deviation of 12.8. Orthodox Christians and the Amhara ethnic group accounted for 89.3% and 94% of the respondents respectively. More than three fourth (76.6%) of the respondents were urban by resident. Two hundred thirty three (60.7%) reported to be married and 93(24.2%) were merchants. For two hundred thirty three (60.7%), it takes half an hour and above on single trip to reach to the hospital. (Table 1)

**Table 1:** Socio demographic characteristic of the study participants (n = 384), GUH, Northwest Ethiopia, 2011

**Variables**		**Frequency**	**Percent (%)**
Sex	Male	142	37.0
	Female	242	63.0
Age	18‐40	36	9.4
	41‐60	210	54.7
	> = 60	138	35.9
Ethnicity	Amhara	361	94.0
	Tigrie	18	4.7
	Others	5	1.3
Residence	Rural	90	23.4
	Urban	294	76.6
Marital status	Single	17	4.4
	Married	233	60.7
	Divorced	41	10.7
	Widowed	93	24.2
Religion	Orthodox	343	89.3
	Muslim	36	9.4
	Others	5	1.3
Employment status	Government	74	19.3
	Merchant	93	24.2
	Student	8	2.1
	Housewife	87	22.7
	Farmer	43	11.2
	Retired	79	20.6
Educational status	Cannot read and write	196	51
	Grade 1‐6	60	15.6
	Grade 7‐12	83	21.6
	Diploma and above	45	11.7
Monthly income	<500 ETB	248	64.6
	500-1000ETB	65	16.9
	> = 1000ETB	71	18.5
Distance from the hospital(single trip)	<0.5 h	151	39.3
	> 0.5 h	233	60.7

### **Clinical, medication and adherence characteristics**

Near half (47.4%) of the respondents had no any of the co morbidities like heart disease, diabetes mellitus, renal diseases and others. More than half (53.4%) of the study subjects had uncontrolled blood pressure. Regarding the medication information, 154 (40.1%) of the subjects have been taking only one drug while 45.3% and 14.6% of them were on two and three and above (≥3) drugs, respectively. Regarding the cost of medication, 59.6% of the respondents were getting their medication for free. In addition, data were also collected on the duration of treatment. Accordingly, 135 (35.2%) of them have been taking their treatment for the last 2 to 4 years. The mean number of pills taken per day was 2.5 with standard deviation of 1.4(Table 2).

**Table 2:** Medication and Blood pressure characteristic of respondents, GUH, Northwest Ethiopia, 2011

**Variables**	**Frequency**	**Percent**
**Number of co morbidity**		
None	182	47.4
1	170	44.3
> = 2	32	8.3
**Blood pressure:**		
Controlled	179	46.6
Uncontrolled	205	53.4
**Number of drugs:**		
One	154	40.1
Two	174	45.3
Three and above	56	14.6
**Dosage:**		
Once daily	179	46.6
BID	196	51.0
TID and above	9	2.3
**Number of pills per day:**		
One pill	112	29.2
Two pills	99	25.8
Three pills	101	26.3
Four and above pills	72	18.8
**Who covered the cost of the drug?**		
Myself	128	33.3
Family	12	3.2
Free	229	59.6
Employer organization	15	3.9
**Duration of treatment in yrs:**		
0.25‐1 yrs	109	28.4
2‐4 yrs	135	35.2
5‐7 yrs	55	14.3
8‐10 yrs	42	10.9
≥ 11 yrs	43	11.2

Only 64.6% of the study subjects were found to be adherent to their treatment. Majority of the respondents (76.8%) were knowledgeable about HTN and its treatment.

### **Factors associated with antihypertensive treatment adherence**

The association of selected socio-demographic, clinical and other characteristics on adherence status was investigated using both the bivariate and multivariate logistic regression technique. Accordingly, variables considered in the bivariate analysis were: age, sex, residence, marital status, religion, educational status, income, employment status, number of co-morbidities, BP level, distance, dosage frequency, duration of treatment, number of medications, coverage of drug cost, knowledge about HTN and Patient provider relationship. Explanatory variables with p value up to 0.2 were included in the multiple logistic regressions. Finally, sex, distance < 0.5 h from the hospital, having no or one co-morbidity, having controlled BP, being knowledgeable about HTN remained to be significantly associated with adherence to treatment of HTN. (Table 3)

**Table 3:** Effect of selected socio-demographic, clinical and other characteristics on adherence to antihypertensive treatment, GUH, Northwest Ethiopia, 2011

Variables		Adherence Status		COR(95%CI)	AOR(95%CI)
		Adheren	Non adherent		
Sex	Male	87	55	0.80(0.51, 1.22)	0.48(0.28, 0.82)**
	Female	161	81	1	1
Residence	Rural	51	39	1	
	Urban	197	97	1.55(0.96, 2.52)*	
Marital Status	Single	13	4	1	
	Married	157	76	0.64	(0.20, 2.02)*
	Divorced	22	19	0.36	(0.10, 1.28)*
	Widowed	56	37	0.47	(0.14, 1.52)*
Religion	Orthodox	226	117	0.60(.31, 1.15)*	
	Others	22	19	1	
Educational status	Illiterate	119	77	1	
	Grade 1-6th	39	21	1.20(0.66, 2.20)*	
	Grade 7-12th	58	25	1.50(0.87, 2.60)*	
	Diploma and above	32	13	1.59(0.79, 3.23)*	
Income level	<= 500 ETB	152	96	1	
	501‐100 ETB	39	26	0.95(0.54, 1.66)*	
	> = 1001 ETB	57	14	2.57(1.36, 4.87)*	
	Government worker	56	18	1.80(0.90, 3.64)*	
	Merchant	54	39	0.8(0.43, 1.49)*	
Employment status	Student	6	2	1.74(0.33, 9.19)*	
	House wife	57	30	1.10(0.58, 2.08)*	
	Farmer	25	18	0.81 (0.38, 1.72)*	
	Retired	50	29		
No of co morbidities	None	118	64	2.09(0.98, 4.46)	2.50(1.01, 6.21)**
	One	115	55	2.37 (1.10, 5.09)	2.68(1.07, 6.71)**
	Two and above	15	17	1	**1**
BP level	Controlled	136	43	2.63(1.69, 4.08)	2.93(1.73, 4.96)**
	Uncontrolled	112	93	1	**1**
Distance in hrs	<0.5 hrs	112	39	2.05(1.31, 3.21)	2.02(1.19, 3.43)**
	≥0.5 hrs	136	97	1	**1**
Dosage frequency	Once daily	123	56	1.10 (0.27, 4.55)*	
	BID	119	77	0.77(0.19,3.18)*	
	TID and above	6	3	1	
Knowledge towards	Knowledgeable	224	71	8.55 (4.99, 14.65)	6.21(3.22, 11.97)**
HTN and its treatment	Not Knowledgeable	24	65	1	**1**
Attitude	Favourable	236	96	8.19 (4.12, 16.29)	3.23(1.31, 7.97)**
	Not favourable	12	40	1	**1**
Pt-provider relationship	Good	239	124	2.57( 1.05, 6.27)*	
	Poor	9	12	1	

Note: - ** statistically significant * variables with p value of greater than 0.2 in crude analysis omitted from entering in to the model.

The multivariate logistic regression showed that as the distance from the hospital decreased, the adherence to treatment of HTN got improved (AOR = 2.02, 95% CI = 1.19, 3.43). Those who have controlled HTN had a significantly higher chance of being adherent to their treatment (AOR = 2.93, 95% CI (1.73, 4.96). The odds of adherence to anti-HTN treatment among knowledgeable Clients was 6 times (AOR = 6.21, 95%CI = 3.22, 11.97) higher than the odds of adherence among HTN patients who were not knowledgeable. The odds of adherence among study participants with no or one co-morbidity were 2.5 and 2.68 times higher than the odds adherence among those who had two co-morbidities (adjusted OR = 2.50, 95%CI 1.01, 6.21) or more than two co-morbidities (adjusted OR = 2.68, 95%CI 1.07, 6.71), respectively.

## **Discussion**

Ensuring patients’ adherence to anti hypertension medications to prevent complications of hypertension remains a major challenge to public health in many developing countries. Poor adherence to treatment is the single most important reason for uncontrolled hypertension, serious complications and wastage of health care resources. Several factors, which may be patient or health system related, continue to militate against compliance behavior [9,13].

Only 64.6% of the study subjects were found to be adherent to their treatment. It is higher than what has been reported from Malaysia (44.2%) and Gambia 27% [10,14]. The adherence to antihypertensive treatment found in the current study was also higher than that of 57% found in the study done in Pakistan [15]. This difference is possibly because more than half (59.6%) of the patients in the current study receive free medical care and drugs whereas in the other study patients had to pay for their treatment. However, it is lower than the studies done in Egypt (74.1%), another part of Pakistan (77%) and Scotland (91%) [8,12,16]. This might be due to better access and care to patients in these countries. It is also supported by the findings of this study that, for 71.3% of the non adherents, the hypertension treatment and care service was not accessible.

In this study, significant association between sex and adherence level was observed. Accordingly, men were found to be less adherent when compared to women. This finding is in line with a study done in India [12], where men had almost threefold increase in risk of non-adherence as compared to women. This can be explained by the fact that; men are burdened by the outdoor activities which make them busy and make them forget their medications. Alcohol consumption, a commonly practice by males, could also be a barrier for their treatment adherence.

Longer distance was a big barrier for adherence to treatment recommendations especially when it was accompanied by poor infrastructure (e.g. lack of transportation) and poverty. Distance from the hospital was another variable found to be significantly and independently associated with the adherence status of the respondents. Those patients from distant areas were less likely to be adherent as compared to study subjects who are closer. This finding is supported by the study done in India [12]. Patients who take long to come to the clinic have certain characteristics that promote non-adherence, which also delayed them from coming for review and possibly collecting drugs from the hospital when they refill the antihypertensive.

Co-morbidities can worsen the conditions of the patient and make them unable to adhere to their antihypertensive medications. This study revealed that the numbers of co morbidities among HTN patients had significant associations on adherence behavior. Patients with no and one co morbidities were more likely to adhere to their treatment than those with two and above co morbidities. Patients with more number of co morbidities could suffer from serious complications and complex treatment regimens which were favorable conditions not to adhere to their medications.

Right knowledge about HTN and its treatment creates a clear understanding and avoids confusion about the treatment and the disease condition. Knowledge about HTN and its treatment was found to be positively associated with adherence behavior. Patients with better awareness were more likely to adhere to their treatment. A similar study from Pakistan and Gaza demonstrated that patients who were aware of their diseases and treatments had better adherence compared to those who did not [17]. However, in contrast to this finding, studies from some developed world indicated no association between knowledge and adherence [18,19].

In this study, blood pressure control level was associated with adherence behavior. Those with controlled blood pressure were observed to be adherent. This finding is in line with the study done in Greece [19]. It might be attributable to better outcome of the treatment, may offer the patient good satisfactions and creates strong motivation towards the treatment. However, bad outcome (uncontrolled BP) could make the patient hopeless and low satisfaction and hence urged them to stop their treatment.

## **Conclusions**

In conclusion, more than half of the study participants were found to be adherent to their treatment. Factors such as sex, distance from the hospital, number of co morbidities, Knowledge about HTN and its treatment were associated with adherence behavior of patients. Early diagnosis and management of co morbidities, adherence counseling and patient education about the disease and its treatment are important to improve adherence status of patients.

This study have the following limitations: self-reporting was used as the only method of measuring adherence. This method has the disadvantages of recall bias and eliciting only socially acceptable responses and hence, may overestimate the level of adherence. In addition, it did not consider HTN patients who did not visit the hospital during the time of the study. Hence, the extent of generalisability is limited only to those similar patients who are on chronic illness follow up care.

## **Competing interests**

The authors declare that they have no competing interests.

## **Authors’ contributions**

AD wrote the proposal, participated in data collection, analyzed the data and drafted the paper. GA, SM and ZB approved the proposal with some revisions, participated in data analysis and revised subsequent drafts of the paper. All authors read and approved the final manuscript.

## Pre-publication history

The pre-publication history for this paper can be accessed here:

http://www.biomedcentral.com/1471-2458/12/282/prepub
